# Supplementary material for: Cancer cells impair monocyte-mediated T cell stimulation to evade immunity
Source: Nature. 2024 Nov 27;637(8046):716–25. doi: 10.1038/s41586-024-08257-4 (PMC7617236; doi:10.1038/s41586-024-08257-4)
Supplement: Supplementary file 1 — Uncropped western blot gel images. [file 41586_2024_8257_MOESM1_ESM.pdf]

nature portfolio

https://doi.org/10.1038/s41586-024-08257-4

Supplementary information

Cancer cells impair monocyte-mediated T cell stimulation to evade immunity

In the format provided by the authors and unedited

UNCROPPED WESTERN BLOT IMAGES

1. A375 Melanoma BLOT: **Extended Data Fig. 5s**

   - HISTONE3 (18kDa)

Cropped area

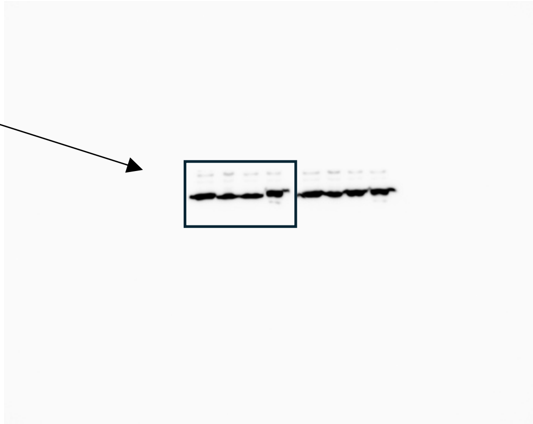

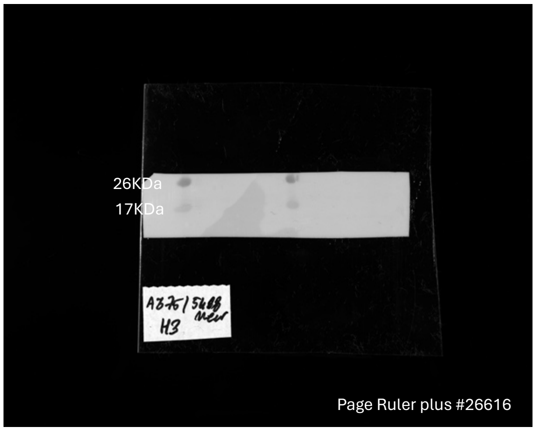

   - COX2 (74 kDa)

Cropped area

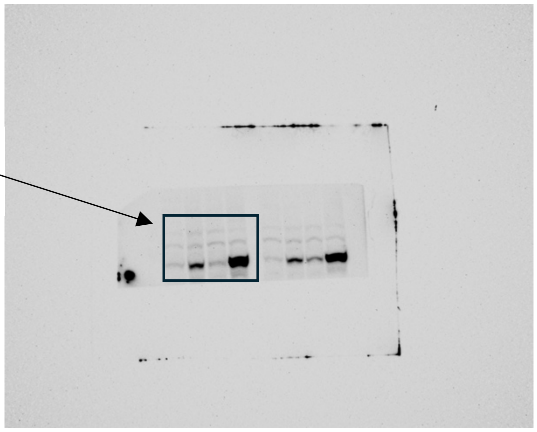

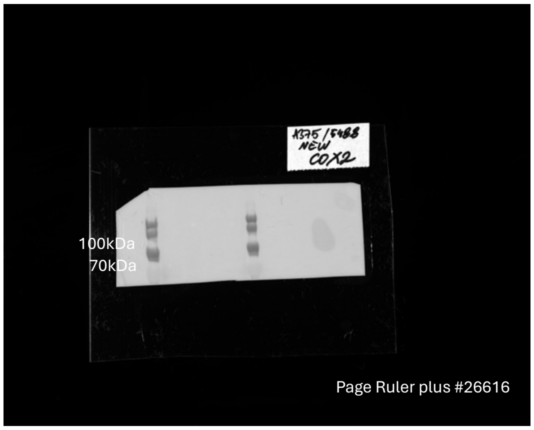

Histone control run on the same gel. Gel was cut for antibody incubation and development.

2. YUMM1.7 Melanoma BLOT: **Extended Data Fig. 5m**

- Vinculin (~116kDa)
  *on left side for COX2 samples on right side for COX1 samples

Cropped area

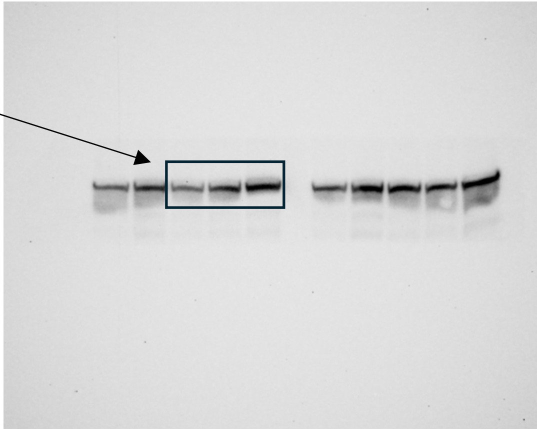

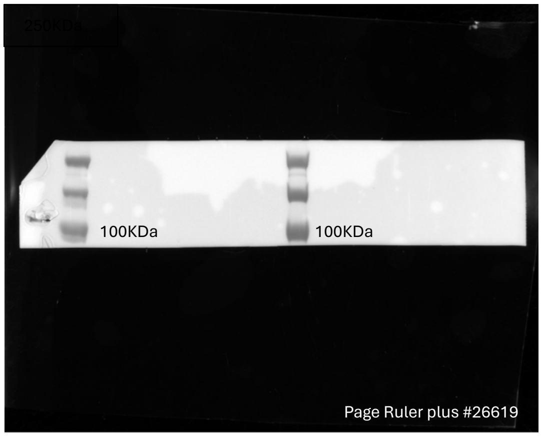

- COX 1 (left) COX2 (right) ~74KDa

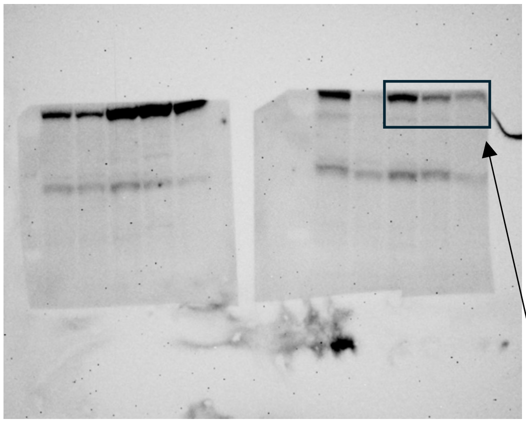

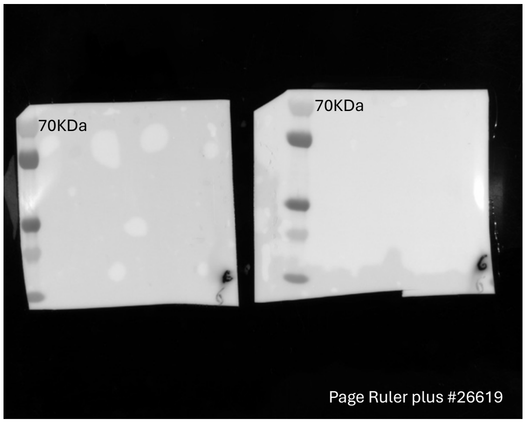

Cropped area

Vinculin control run on the same gel. Gel was cut for antibody incubation and development.
