# Supplementary material for: Cancer cells impair monocyte-mediated T cell stimulation to evade immunity
Source: Nature. 2024 Nov 27;637(8046):716–25. doi: 10.1038/s41586-024-08257-4 (PMC7617236; doi:10.1038/s41586-024-08257-4)
Supplement: Supplementary file 2 — Reporting Summary [file 41586_2024_8257_MOESM2_ESM.pdf]

nature portfolio

Corresponding author(s): Anna C. Obenauf

Last updated by author(s): 25.09.2024

Reporting Summary

Nature Portfolio wishes to improve the reproducibility of the work that we publish. This form provides structure for consistency and transparency in reporting. For further information on Nature Portfolio policies, see our Editorial Policies and the Editorial Policy Checklist.

Statistics

For all statistical analyses, confirm that the following items are present in the figure legend, table legend, main text, or Methods section.

n/a	Confirmed	
☐	☒	The exact sample size (*n*) for each experimental group/condition, given as a discrete number and unit of measurement
☐	☒	A statement on whether measurements were taken from distinct samples or whether the same sample was measured repeatedly
☐	☒	The statistical test(s) used AND whether they are one- or two-sided<br>*Only common tests should be described solely by name; describe more complex techniques in the Methods section.*
☐	☒	A description of all covariates tested
☐	☒	A description of any assumptions or corrections, such as tests of normality and adjustment for multiple comparisons
☐	☒	A full description of the statistical parameters including central tendency (e.g. means) or other basic estimates (e.g. regression coefficient) AND variation (e.g. standard deviation) or associated estimates of uncertainty (e.g. confidence intervals)
☐	☒	For null hypothesis testing, the test statistic (e.g. *F*, *t*, *r*) with confidence intervals, effect sizes, degrees of freedom and *P* value noted<br>*Give P values as exact values whenever suitable.*
☒	☐	For Bayesian analysis, information on the choice of priors and Markov chain Monte Carlo settings
☒	☐	For hierarchical and complex designs, identification of the appropriate level for tests and full reporting of outcomes
☐	☒	Estimates of effect sizes (e.g. Cohen's *d*, Pearson's *r*), indicating how they were calculated

Our web collection on statistics for biologists contains articles on many of the points above.

Software and code

Policy information about availability of computer code

Data collection: Flow cytometry data was acquired using an LSR Fortessa (BD) or BD Aria. Immunofluorescence staining's were imaged on a TCS SP8 confocal microscope (Leica). For tracking bioluminescence signal from transferred T cells an IVIS imager (Perkin Elmer) was used. Single-cell RNA-seq libraries were sequenced on NovaSeq (Illumina). Western blot images were acquired on a ChemiDoc Imaging system (BioRad). qPCR data was obtained using a CFX384 Real-Time-Cycler (BioRad).

Data analysis: scRNA-seq analysis TME
CD45+ immune cells were collected in 4 different 10x Genomics sequencing experiments: Exp1-Chromium Single Cell 3' scRNA-seq samples pre-processed using cellranger count v6.1.1 (Y3.3 samples: NTT/108155 and RTT/108157); Exp2- 3' CellPlex multiplex experiment with 4 samples pre-processed using cellranger multi v6.1.1 (YUMM1.7OVA samples: NTT_3A6_ACT, RTT_2B12_ACT, RTT_PTGS12KO_ACT, RTT_ROSA26_ACT). Exp3 and Exp4-Chromium Flex multiplex experiments with 4 samples each pre-processed using cellranger multi v7.1.0 and the build in Probe Set v1.0.1 mm10-2020-A. Exp3- YUMM1.7OVA samples: RTT mCherry CTRL, RTT IRF3/7, RTT COX2i and RTT COX2i + 5-AZA; Exp4- YUMM1.7OVA contained biological replicates of Exp2 samples and untreated YUMM1.7OVA samples (noA): NTTnoA/271221, RTTnoA/271222, NTT/271223, RTT/271224). The prebuilt 10X mm10 reference refdata-gex-mm10-2020-A was used. Further processing was performed in R v4.2.2 with Seurat v4.3.0. For generating a CD45+ immune reference map we integrated cells from the first three experiments as follows. The cellranger filtered feature-barcode matrices were used, retaining cells with more than 1000 detected genes and less than 15% of mitochondrial and less than 40% of ribosomal RNA reads. An integrated feature-barcode matrix from the 3 experimental batches was generated accounting for the inclusion of a probe-based assay by keeping genes found in at least 5 cells in each experiment, and excluding ribosomal and mitochondrial genes. Data was log-normalized, scaled (regressing out the difference between the G2M and S phase signature scores), dimensionality reduction was performed using PCA on the top 3000 most variable genes, batch correction across batches was performed using Harmony84 v0.1.1, the 40 harmony embeddings were used for UMAP visualizations. The first 40 harmony dimensions were used to identify immune cell subclusters with a resolution of 0.5, that were further assigned to cell types using known markers and publicly

available myeloid reference data-sets. Cells were scored for the expression of published signatures using the AddModuleScore function85. Wilcoxon rank sum test implemented in Presto v1.0.0 was used to identify differentially expressed genes. Seurat's reference-based mapping was used to predict celltype identity and map cells of the biological replicate experiment to our annotated reference set using the FindTransferAnchors and MapQuery functions, after a quality control process retaining cells between 1000 and 4500 detected genes for 271222 and 271224, and 1300 and 8000 detected genes for 271221 and 271223, and limiting count tables to the gene universe of the reference. Depth normalized counts for pseudobulk and GSEA functional analyses of this experiment were generated using cellranger aggr. Differences between ACT and untreated conditions (no ACT) from the replicate experiment (Exp4) were explored on a pseudo-bulk level in an unsupervised clustering analysis with heatmap visualization. The fibroblast cluster was removed prior to further processing. Sum aggregation on the depth-normalized UMI counts, was followed by variance stabilizing transformation, selection of 300 most variable genes, standardization, kmeans clustering (k=3) and enrichr analysis against the Reactome_2022 using enrichR.

scRNA-seq analysis intratumoral CD8+ OT-1 T cells
Single-cell gene-expression of isolated NTT and RTT T cells was assayed in a Chromium Flex experiment, read processing was performed using cellranger multi v7.1.0 using probeset v1.0.1 mm10-2020-A. Cellranger filtered feature-barcode matrices were used on, further filtered to-retain cells with more than 800 detected genes, less than 10 percent of mitochondrial and less than 10 percent of ribosomal RNAs reads and remove cells of contaminant clusters identified using SingleR and ImmGen reference (fibroblasts, MoMac populations). Data was log-normalized, scaled, dimensionality reduction was performed using PCA on the top 2000 most variable genes, harmony was used for the integration of cells from different samples, 15 harmony embeddings were used for UMAP visualizations, and published tumor single cell data for signature scoring. Gene lists are deposited in Supplementary Table 2.

RNA velocity analysis
Loom files containing the splicing annotation were created for each sample using the velocyto run command from the package velocyto (0.17,17), with default parameters, with no masked intervals. Then, the loom files were combined with the scRNA-seq object that had been filtered to keep the data for monocyte and macrophage populations (Monocyte_1, Monocyte_2, Infl_Mono, TAM_CCL6, TAM_Ctsk, TAM_C1q, TAM_H2Ab1, TAM_Spp1, TAM_cycling), for each condition (NTT, RTT, RTT PTGS12KO). First and second order moments were computed using scvelo (0.2.5) pp.moments (n_pcs = 30, n_neighbors = 30) and the dynamical model was run with default parameters. Python version 3.8.12 was used.

SCENIC analysis
Gene regulatory networks for each cell population in each condition were calculated using SCENIC48. The motif database used was mm9-tss-centered-10kb-7species.mc9nr.feather. Co-expression network was calculated using GENIE3. The gene regulatory network was built using SCENIC wrapper functions.

TME signatures in immunotherapy-treated human samples
Gene expression data for patients receiving checkpoint blockade were obtained from Gide et al. (accession no. PRJEB23709). The TME-COX, TME-IRF3/7 and CD8+ T cell scores for each tumor sample were defined as the geometric mean of the expression values of each of the gene sets, respectively (Supplementary Table 4). Univariate Cox proportional hazards models, in which the TME-COX and TME-IRF3/7 scores were included as continuous variables, were used for testing the statistical association between gene signature expression and patient survival, separately for both signatures. The tumor samples were then divided into three groups based on the signature score (bottom third, mid third, top third) and Kaplan-Meier plots were generated for visualization. The association between signature expression and CD8+ T cell abundance was evaluated by calculating the Person's correlation coefficient between the signature score and a CD8+ score for each signature separately. For this all scores were normalized to a median of zero and standard deviation of one. The two overlapping genes were removed from the CD8+ signature before comparing it to TME-IRF3/7 signature expression. For evaluating the enrichment of TME-COX and TME-IRF37 gene signatures in responder and non-responder patients to TIL therapy (baseline) from Barras et al. mouse gene ids were first converted to human orthologs (with DIOPT v9; Best Score = Yes, Best Score Reverse = Yes, DIOPT Score > 7) and single-cell level signature enrichment scores for the "humanized" gene sets were calculated using AddModuleScore_UCell.

For acquisition of flowcytometry data the FACS DIVA software (v9.0.1) was used. For analysis of flowcytometry data we used Flowjo (version 10.8 or newer).
Statistical analysis was performed with Graphpad Prism (v9.1.2 or newer) or Microsoft Excel (v 16.88)
Immunofluorescence images were adaptively deconvoluted using the Leica TCS SP8 LIGHTNING tool v3.5.7.23225 and further analyzed using Imaris (v9.9).
Metabolites from the untargeted metabolomics approach were annotated using the compound discoverer software (v3.0).
Bioluminescence signal to track T cell infiltration was analyzed using the Living Image software (v4.4)

For manuscripts utilizing custom algorithms or software that are central to the research but not yet described in published literature, software must be made available to editors and reviewers. We strongly encourage code deposition in a community repository (e.g. GitHub). See the Nature Portfolio guidelines for submitting code & software for further information.

Data

Policy information about availability of data

All manuscripts must include a data availability statement. This statement should provide the following information, where applicable:

- Accession codes, unique identifiers, or web links for publicly available datasets
- A description of any restrictions on data availability
- For clinical datasets or third party data, please ensure that the statement adheres to our policy

Gene expression data from YUMM1.7 NTT and RTT cancer cells sorted from tumors was previously deposited under the Gene Expression Omnibus accession number GSE132443. Specifically samples from NTT tumors (GSM3864154,GSM3864155, GSM3864157) and RTT tumors (GSM3864170, GSM3864172,GSM3864173) were used to generate plots in Fig. 3a and Extended Data Fig. 5i. Raw and processed files of the single-cell RNA sequencing generated in this study have been deposited under the GEO accession number GSE241750. Expression data from the Gide et al. study is publicly available (accession no. PRJEB23709). Single cell RNA data from the Barras et al. study, including the Seurat object for the myeloid compartment was obtained upon direct request. Expression data from the Cheng et al. study was publicly available (GSE154763) and the raw count matrix was obtained upon request. Source data is provided for all graphs that are shown in the manuscript. All other formats of raw data are available upon request.

Research involving human participants, their data, or biological material

Policy information about studies with human participants or human data. See also policy information about sex, gender (identity/presentation), and sexual orientation and race, ethnicity and racism.

Reporting on sex and gender	Biopsies were obtained from patients with an age at diagnosis that ranged from 24 to 85 with a median of 66. 34% were female patients and 66% were male patients.
Reporting on race, ethnicity, or other socially relevant groupings	*Please specify the socially constructed or socially relevant categorization variable(s) used in your manuscript and explain why they were used. Please note that such variables should not be used as proxies for other socially constructed/relevant variables (for example, race or ethnicity should not be used as a proxy for socioeconomic status).* *Provide clear definitions of the relevant terms used, how they were provided (by the participants/respondents, the researchers, or third parties), and the method(s) used to classify people into the different categories (e.g. self-report, census or administrative data, social media data, etc.)* *Please provide details about how you controlled for confounding variables in your analyses.*
Population characteristics	We obtained cell-segmented data for 74 FOVs (Field of View, an area of 500x500 µm) from TMA cores of 34 melanoma metastases, in total consisting of 980 genes x 171,536 cells. Tumor tissues were obtained from 21 lymph nodes, 7 subcutaneous metastases, 1 lung metastasis and 1 brain metastasis (4 NAs), from 31 patients containing 72 FOVs. 2 FOVs were from tonsils as control. Most tumor tissue were from patients that were treatment naïve at the time of surgery. Tissue collection was approved by the Regional Ethics committee at Lund University (Dnr. 191/2007 and 101/2013). Patients signed an informed consent. The majority of TMA cores contained tertiary lymphoid structures (TLS) and FOVs were preferentially directed to these regions. Low-quality FOVs, cells with < 20 counts and potential multiplets of cells (area exceeding the sample geometric mean + 5 standard deviation) were discarded.
Recruitment	*Describe how participants were recruited. Outline any potential self-selection bias or other biases that may be present and how these are likely to impact results.*
Ethics oversight	Tissue collection was approved by the Regional Ethics committee at Lund University (Dnr. 191/2007 and 101/2013). Patients signed an informed consent.

Note that full information on the approval of the study protocol must also be provided in the manuscript.

Field-specific reporting

Please select the one below that is the best fit for your research. If you are not sure, read the appropriate sections before making your selection.

☒ Life sciences ☐ Behavioural & social sciences ☐ Ecological, evolutionary & environmental sciences

For a reference copy of the document with all sections, see nature.com/documents/nr-reporting-summary-flat.pdf

Life sciences study design

All studies must disclose on these points even when the disclosure is negative.

Sample size	The effect size was determined from prior research or pilot experiments relevant to our study, especially from Haas et al., 2021. This estimate informed our power analysis and allowed us to choose a sample size that would balance statistical power with practical considerations. The statistical tests planned for the analysis were considered when determining sample size. Practical considerations such as time, cost, and resource availability were also considered. Ethical considerations (3R) played a role in determining sample sizes for in vivo experiments. We aimed to use the smallest number of subjects necessary in in vivo studies to achieve reliable and valid results.
Data exclusions	No data points were excluded, except for when tumors became necrotic as this is an end-point in our current working animal license or when mice displayed weight loss and sickness and had to be euthanized.
Replication	All in vitro and in vivo experiments were repeated at least twice and always with multiple replicates, except for the following experiments which were performed only once: scRNA-seq of experiments involving pharmacological treatment of the YUMM1.7 RTT model, intratumoral injection of T cells and the YUMM3.3 model. IF stainings for which representative images are shown were repeated at least twice, except for the NTT in Batf3-/- and Nur77 reporter experiment which was performed once but with n=3 tumors and was also confirmed with flow cytometry. Pharmacological combination treatments of the KPAR model were performed once.
Randomization	Mice were allocated to treatment or control group based on the average tumor size to achieve similar tumor burden between groups at the time of treatment start. For experiments where drug treatment had to be started at day 3 post injection, mice were randomly allocated to treatment groups. Within an experiment age and sex-matched mice were used. For all other experiments samples were randomly allocated.
Blinding	Scientists were not blinded to genotypes or treated groups prior to data collection and during analysis in order to prevent mislabeling of genotypes and treatment groups. Experiments were repeated by different independent investigators.

Reporting for specific materials, systems and methods

We require information from authors about some types of materials, experimental systems and methods used in many studies. Here, indicate whether each material, system or method listed is relevant to your study. If you are not sure if a list item applies to your research, read the appropriate section before selecting a response.

Materials & experimental systems

n/a	Involved in the study
☐	☒ Antibodies
☐	☒ Eukaryotic cell lines
☒	☐ Palaeontology and archaeology
☐	☒ Animals and other organisms
☒	☐ Clinical data
☒	☐ Dual use research of concern
☒	☐ Plants

Methods

n/a	Involved in the study
☒	☐ ChIP-seq
☐	☒ Flow cytometry
☒	☐ MRI-based neuroimaging

Antibodies

Antibodies used

Antibodies for flowcytometry
anti-mouse AXL PE-Cy7 eBioscience Cat#25-1084-82, clone: MAXL8DS 1:200
anti-mouse CD103 PerCP/Cyanine5.5 BioLegend Cat#121415 , clone: 2E7 1:100
anti-mouse CD103 PE BioLegend Cat#121405 , clone: 2E7 1:100
anti-mouse CD11b APC eBioscience Cat#17-0112-81, clone: M1/70 1:200
anti-mouse CD11b PerCP/Cyanine5.5 BioLegend Cat#101229, clone: M1/70 1:200
anti-mouse CD11c BV605 BD Pharmingen Cat#563057, clone: HL3 1:100
anti-mouse CD11c FITC BioLegend Cat#117305, clone: N418 1:100
anti-mouse CD16/CD32 (Mouse BD Fc Block) BD Pharmigen Cat#553141, clone: 2.4G2 1:50
anti-mouse CD24 BV510 BioLegend Cat#101831, clone: M1/69 1:100
anti-mouse CD24 FITC eBioscience Cat#11-0242-82, clone: M1/69 1:100
anti- mouse CD279/PD-1 BV785 BioLegend Cat#135225, clone: 29F.1A12 1:200
anti-mouse CD279/PD-1 FITC BioLegend Cat#135213, clone: 29F.1A12 1:200
anti-mouse CD40 APC BioLegend Cat#124611, clone: 3/23 1:200
anti-mouse CD45 BV711 BioLegend Cat#103147, clone: 30-F11 1:500
anti-mouse CD45 FITC BioLegend Cat#103107, clone:30-F11 1:500
anti-mouse CD86 BV510 BioLegend Cat#105039, clone: GL-1 1:100
anti-mouse CD3 BV605 BD Horizon Cat#564009, clone:17A2 1:100
anti- mouse CD3 AF647 BioLegend Cat#100209, clone:17A2 1:100
anti-mouse CD3 AF488 BioLegend Cat#100212, clone:17A2 1:100
anti-mouse CD8a eFluor 450 eBioscience Cat#48-0081-80, clone:53-6.7 1:100
anti-mouse CD8a AF594 BioLegend Cat#100758, clone:53-6.7 1:100
anti-mouse Ly-6C BV785 BioLegend Cat#128041, clone:HK1.4 1:100
anti-mouse MHCI (H-2Kb) APC eBioscience Cat#17-5958-82,clone:AF6-88.5.5.3 1:200
anti-mouse MHCI (H2Kb) PE eBioscience Cat#12-5958-80, clone:AF6-88.5.5.3 1:200
anti-mouse MHCII (I-A/I-E) eFluor450 eBioscience Cat#48-5321-80, clone:M5/114.15.2 1:200
anti-mouse I-A/I-E APC BioLegend Cat#107613, clone:M5/114.15.2 1:200
anti-mouse NK-1.1 BV711 BioLegend Cat#108745, clone: PK136 1:100
anti-mouse TCF1 PE BD Pharmingen Cat#564217, clone: S33-966 1:50
anti-mouse TIM3 BV711 BioLegend Cat#119727, clone: RMT3-23 1:100
anti-mouse CD88 PE BioLegend Cat#135805, clone: 20/70 1:100
anti-mouse Ly-6A/E (Sca-1) FITC BioLegend Cat#108105, clone: D7 1:100
anti-mouse SIINFEKL-HK2B PE Invitrogen Cat#12-5743-81, clone: 25-D1.16 1:100
anti-mouse F4/80 PE BioLegend Cat#B123110, clone: BM8 1:200
Rat IgG1, K Isotype control PE BD Pharmingen Cat#5546, clone: R3-34

Antibodies for T cell activation
CD3e Monoclonal Antibody, functional grade eBioscience Cat#16-0031-81, clone: 145-2C11
CD28 Monoclonal Antibody, functional grade eBioscience Cat#16-0281-81, clone: 36.51

Antibodies for in vivo treatment
InVivoMAb anti-mouse PD-1 (CD279) BioXCell Cat#BE0146, clone:RMP1-14
InVivoMAb anti-mouse CTLA-4 (CD152) BioXCell Cat#BE0164, clone:9D9
InVivoMAb anti-mouse NK1.1 BioXCell Cat#BE0036, clone:PK136
Monoclonal antibody anti-mouse CD8 in house produced clone: 2.43
InVivoMAb anti-mouse IFNAR-1 BioXCell Cat#BE0241, clone: MAR1-5A3
InVivoMAb anti-mouse IFNg BioXCell Cat#BE0055, clone: XMG1.2
InVivoMAb recombinant Flt-3L-Ig BioXCell Cat#BE0098, clone: hum/hum
InVivoMAb rat IgG2b isotype control BioXCell Cat#BE0090, clone: LTF2
InVivoMAb rat IgG2a isotype control BioXCell Cat#BE0089, clone: 2A3
InVivoMAb mouse IgG1 isotype control BioXCell Cat#BE0083, clone: MOPC-21

Antibodies for western blot
anti-mouse Vinculin mAb Sigma Aldrich Cat#V9131, hVIN-1

anti-mouse COX2 mAb Cell Signaling Technology Cat# 12282S, D5H5
anti-rabbit IgG HRP-linked Cell Signaling Technology Cat#7074
anti-mouse IgG HRP-linked Cell Signaling Technology Cat#7076

Antibodies for in vitro treatment
InVivoMAb anti-mouse IFNAR-1 BioXCell Cat#BE0241, clone: MAR1-5A3
InVivoMAb mouse IgG1 isotype control BioXCell Cat#BE0083, clone: MOPC-21

Antibodies for IF staining
anti-mouse CD3 BV421 Biolegend Cat#100227, clone 17A2
anti-mouse MHCII (I-A/I-E) BV510 Biolegend Cat#107635, clone M5/114.15.2
anti-mouse CD103 unconjugated R&D Systems Cat#AF1990, polyclonal
anti-mouse FSCN1 AF594 SCBT Cat#sc-21743, clone 55-k2
anti-mouse LY6C AF647 Biolegend Cat#128010, clone HK1.4

Validation

Antibodies for flowcytometry
https://www.thermofisher.com/antibody/product/Axl-Antibody-clone-MAXL8DS-Monoclonal/25-1084-82
https://www.biolegend.com/en-us/products/percp-cyanine5-5-anti-mouse-cd103-antibody-5599
https://www.biolegend.com/en-us/products/pe-anti-mouse-cd103-antibody-3574
https://www.thermofisher.com/antibody/product/CD11b-Antibody-clone-M1-70-Monoclonal/17-0112-82
https://www.biolegend.com/en-us/products/percp-anti-mouse-human-cd11b-antibody-4315
https://www.bdbiosciences.com/en-at/products/reagents/flow-cytometry-reagents/research-reagents/single-color-antibodies-ruo/bv605-hamster-anti-mouse-cd11c.563057
https://www.biolegend.com/en-us/search-results/fitc-anti-mouse-cd11c-antibody-1815?GroupID=BLG11937&gclid=Cj0KCQjwi7GnBhDXARIsAFLvH4mVVU5ae5X-Cx6DF9ti4yolV5-O__oZQAqSb2y-Dr5SkTcc4O_-saAaAgb2EALw_wcB
https://www.bdbiosciences.com/en-at/products/reagents/flow-cytometry-reagents/research-reagents/single-color-antibodies-ruo/purified-rat-anti-mouse-cd16-cd32-mouse-bd-fc-block.553141
https://www.biolegend.com/de-at/products/brilliant-violet-510-anti-mouse-cd24-antibody-9925
https://www.thermofisher.com/antibody/product/CD24-Antibody-clone-M1-69-Monoclonal/11-0242-82
https://www.biolegend.com/en-us/products/brilliant-violet-785-anti-mouse-cd279-pd-1-antibody-9874
https://www.biolegend.com/en-us/products/fitc-anti-mouse-cd279-pd-1-antibody-7004
https://www.biolegend.com/de-at/products/apc-anti-mouse-cd40-antibody-4984
https://www.biolegend.com/de-at/products/brilliant-violet-711-anti-mouse-cd45-antibody-10439
https://www.biolegend.com/de-at/products/fitc-anti-mouse-cd45-antibody-99
https://www.biolegend.com/de-at/products/brilliant-violet-510-anti-mouse-cd86-antibody-8745
https://www.biolegend.com/en-gb/products/brilliant-violet-605-anti-mouse-cd3-antibody-8503
https://www.biolegend.com/en-gb/products/alexa-fluor-647-anti-mouse-cd3-antibody-2693
https://www.biolegend.com/en-gb/products/alexa-fluor-488-anti-mouse-cd3-antibody-2835
https://www.thermofisher.com/antibody/product/CD8a-Antibody-clone-53-6-7-Monoclonal/48-0081-82
https://www.biolegend.com/en-gb/products/alexa-fluor-594-anti-mouse-cd8a-antibody-9608
https://www.biolegend.com/en-gb/products/brilliant-violet-785-anti-mouse-ly-6c-antibody-11982
https://www.thermofisher.com/antibody/product/MHC-Class-I-H-2Kb-Antibody-clone-AF6-88-5-5-3-Monoclonal/17-5958-82
https://www.thermofisher.com/antibody/product/MHC-Class-I-H-2Kb-Antibody-clone-AF6-88-5-5-3-Monoclonal/12-5958-82
https://www.thermofisher.com/antibody/product/MHC-Class-II-I-A-I-E-Antibody-clone-M5-114-15-2-Monoclonal/48-5321-82
https://www.biolegend.com/en-gb/products/apc-anti-mouse-i-a-i-e-antibody-2488
https://www.biolegend.com/en-gb/products/brilliant-violet-711-anti-mouse-nk-1-1-antibody-9576
https://www.bdbiosciences.com/en-at/products/reagents/flow-cytometry-reagents/research-reagents/single-color-antibodies-ruo/pe-mouse-anti-tcf-7-tcf-1.564217
https://www.biolegend.com/en-gb/products/brilliant-violet-711-anti-mouse-cd366-tim-3-antibody-14918
https://www.biolegend.com/en-gb/products/pe-anti-mouse-cd88-c5ar-antibody-6240
https://www.biolegend.com/en-gb/products/fitc-anti-mouse-ly-6a-e-sca-1-antibody-227
https://www.thermofisher.com/antibody/product/OVA257-264-SIINFEKL-peptide-bound-to-H-2Kb-Antibody-clone-eBio25-D1-16-25-D1-16-Monoclonal/12-5743-81
https://www.biozym.com/8667/pe-anti-mouse-f4/80
https://www.bdbiosciences.com/en-at/products/reagents/flow-cytometry-reagents/research-reagents/flow-cytometry-controls-and-lysates/pe-rat-igg1-isotype-control.554685

Antibodies for T cell activation
https://www.thermofisher.com/antibody/product/CD3e-Antibody-clone-145-2C11-Monoclonal/16-0031-82
https://www.thermofisher.com/antibody/product/CD28-Antibody-clone-37-51-Monoclonal/16-0281-82

Antibodies for in vivo treatment
https://bioxcell.com/invivoplus-anti-mouse-pd-1-cd279-bp0146?psafe_param=1&gad=1&gclid=CjwKCAjwrranBhAEEiwAzbhNtaF7E-IyhEPPbzG9YrqPKZ1Erf9_b5hU3drWDF0xA7iwNPdAPrGy1BoCYjAQAvD_BwE
https://bioxcell.com/invivoplus-anti-mouse-ctla-4-cd152-bp0164?gad=1&gclid=CjwKCAjwrranBhAEEiwAzbhNtfZq0JcYygOuO_4HYJRrZQadiKd5ehKt7US_gjQZQ11eC6UR9rhLShoCglkQAvD_BwE
https://bioxcell.com/invivomab-anti-mouse-nk1-1-be0036
https://bioxcell.com/invivomab-anti-mouse-ifnar-1-be0241?psafe_param=1&gad=1&gclid=CjwKCAjwrranBhAEEiwAzbhNtblHOyWh77kCe1_BIoqolfJmLr7FY7mJLFukpKcCXKSxvWJSqpxtLxoC_ZMQAvD_BwE
https://bioxcell.com/invivomab-anti-mouse-ifn-gamma-be0055#tab_pdetails
https://bioxcell.com/invivomab-recombinant-flt-3l-ig-hum-hum
https://shop.bio-connect.nl/antibodies/invivomab-rat-igg2b-isotype-control/be0090_25mg/sfid/5189041

https://www.biozol.de/de/product/bxc-be0089-100mg
https://bioxcell.com/invivomab-mouse-igg1-isotype-control-unknown-specificity-be0083?gad=1&gclid=CjwKCAjwrranBhAEEiwAzbhNtY8dTzxEJQ6To_BBBEXR2fCjoEhd3keXbgLEDPy5Tzxj_EIlQUTgKRoCYYAQAvD_BwE

Antibodies for western blot
https://www.sigmaaldrich.com/AT/de/product/sigma/v9131
https://www.cellsignal.com/products/primary-antibodies/cox2-d5h5-xp-rabbit-mab/12282?_requestid=140729
https://www.cellsignal.com/products/secondary-antibodies/anti-rabbit-igg-hrp-linked-antibody/7074?_requestid=699267
https://www.cellsignal.com/products/secondary-antibodies/anti-mouse-igg-hrp-linked-antibody/7076

Antibodies for in vitro treatment
https://bioxcell.com/invivomab-anti-mouse-ifnar-1-be0241?psafe_param=1&gad=1&gclid=CjwKCAjwrranBhAEEiwAzbhNtblHOyWh77kCe1_BloqolfJmLr7FY7mJLFukpKcCXKSxvWJSqpxtLxoC_ZMQAvD_BwE
https://bioxcell.com/invivomab-mouse-igg1-isotype-control-unknown-specificity-be0083?gad=1&gclid=CjwKCAjwrranBhAEEiwAzbhNtY8dTzxEJQ6To_BBBEXR2fCjoEhd3keXbgLEDPy5Tzxj_EIlQUTgKRoCYYAQAvD_BwE

Antibodies for IF staining
https://www.biolegend.com/en-us/products/brilliant-violet-421-anti-mouse-cd3-antibody-7326
https://www.biolegend.com/en-us/products/brilliant-violet-510-anti-mouse-i-a-i-e-antibody-7997
https://www.rndsystems.com/products/mouse-integrin-alphae-cd103-antibody_af1990
https://www.scbt.com/p/fascin-1-antibody-55k-2
https://www.biolegend.com/en-us/products/alexa-fluor-647-anti-mouse-ly-6c-antibody-4897

Eukaryotic cell lines

Policy information about cell lines and Sex and Gender in Research

Cell line source(s)

Original YUMM1.7 and YUMM3.3 cell-lines were obtained from the Bosenberg Laboratory. All derivatives of these cell-lines were previously generated and described in Haas et al., 2021, Nature Cancer.
A375, M249 and LOX cell-lines and RTT derivatives were obtained from the Massague Laboratory/MSKCC. All derivatives of these cell-lines were previously generated and described in Obenauf et al., 2015, Nature.
NCI-H358 cells were obtained from ATCC and RTT derivatives were generated in house.
CT-26 were purchased from ATCC. All derivatives were previously generated and described in Haas et al., 2021, Nature Cancer.
EPP2 were obtained from the Zuber Laboratory/IMP.
KPAR cell-line was obtained from the Downward Laboratory/CRICK Institute.
HEK-293T cells were purchased from Takara (Lenti-X 293T, 632180).
BLaER-1 cell-line was obtained from the Gaidt Laboratory/IMP
MONO-MAC-1 cell-line was obtained from the Zuber Laboratory/IMP

Authentication

STR Profiling was performed in-house for the YUMM 1.7, YUMM 3.3, EPP2 and KPAR cell lines. Moreover sensitivity to MAPK inhibitors was confirmed for A375, M249 and LOX (BRAFi), CT-26 (MEKi) and for H358 (KRASi).

Mycoplasma contamination

Cells were routinely tested for mycoplasma contamination using our in-house PCR-system. Cells tested negative for mycoplasma contamination.

Commonly misidentified lines
(See ICLAC register)

No commonly misidentified cell lines were used in this study.

Animals and other research organisms

Policy information about studies involving animals; ARRIVE guidelines recommended for reporting animal research, and Sex and Gender in Research

Laboratory animals

All mice were bred and housed in pathogen-free conditions with a housing temperature of 22±1°C, 55 ± 5% humidity, and a photoperiod of 14 hours light and 10 hours dark. Within each experiment, age and sex-matched groups were used. B6.129S(C)-Batf3tm1Kmm/J (Batf3-/-) mice, B6(Cg)-Zbtb46tm1(HBEGF)Mnz/J (zDC-DTR) mice, B6.Cg-Tg(Itgax-cre)1-1Reiz/J (CD11c-Cre) mice and NOD.Cg-Prkdcscid Il2rgtm1Wjl/SzJ (NSG) mice, mice were purchased from Jackson Laboratories. B6.Cg-Rag2tm1.1Cgn/J Ly5.2 (Rag2-/-) mice, BALB/c and C57BL/6J were obtained from the Vienna Biocenter in-house breeding facility. ItgaxCrePtger2−/−Ptger4fl/fl mice were kindly provided by Dr. Jan Boettcher (TUM, Munich). For Rag2-/- Batf3-/- strain generation, Batf3-/- were crossed to Rag2-/- mice and homozygous offspring (Rag2-/- x Batf3-/-) were confirmed by genotyping and used in subsequent experiments to evaluate the lack of cDC1s in the context of adoptive T cell transfer. For Rag2-/- zDC-DTR strain generation, zDC-DTR mice were crossed to Rag2-/- mice and homozygous offspring were confirmed by genotyping and used in subsequent experiments to evaluate the effects of DC depletion. For adoptive T cell transfer experiments and injection of YUMM1.7OVA cell-lines, Rag2-/- mice were used. For the injection of YUMM3.3, KPAR and EPP2 cell-lines C57BL/6 mice were used. For the injection of the CT-26 cell line BALB/c mice were used. For the generation of bone marrow derived dendritic cells (BM-DCs) and Ly6C+ monocytes, bones (femurs and tibias) were collected from in-house-bred C57BL/6 mice. For all strains listed above, mice were used between 6-12 weeks old. For OT-1Luc CD8+ T cell isolations, 6-24 week-old OT-1Luc Thy1.1 mice were used.

Wild animals

Study did not involve wild animals.

Reporting on sex	Within each experiment, sex-matched mice were used whenever possible. Phenotypes and results were confirmed both in male and female mice
Field-collected samples	Study did not involved field-collected samples.
Ethics oversight	All mouse experiments were performed according to our license approved by the Austrian Ministry (GZ: MA58-2260492-2022-22, GZ: 340118/2017/25, BMBWF-66.015/0009-V/3b/2019, GZ: 801161/2018/17 and GZ: 2021-0.524.218 and their amendments.

Note that full information on the approval of the study protocol must also be provided in the manuscript.

Flow Cytometry

Plots

Confirm that:

☒ The axis labels state the marker and fluorochrome used (e.g. CD4-FITC).

☒ The axis scales are clearly visible. Include numbers along axes only for bottom left plot of group (a 'group' is an analysis of identical markers).

☒ All plots are contour plots with outliers or pseudocolor plots.

☒ A numerical value for number of cells or percentage (with statistics) is provided.

Methodology

Sample preparation	For flow cytometry-based characterization of the TME, tumors were isolated between day 7-11 post-injection, cut into pieces, and digested for 1.5 h at 37°C with collagenase A (1 mg/ml, Roche) and DNAse (20µg/ml, Worthington) in unsupplemented RPMI-1640 media. Digested tumors were strained through a 70µm filter and resuspended in FACS buffer (0.5% BSA, 2mM EDTA). FC-block was performed with anti-CD16/32 (clone 2.4G2, BD Pharmingen) for 10 min at 4°C to avoid nonspecific antibody binding, and staining for cell-surface markers was performed for 30 min at 4°C. For intracellular staining the Foxp3 Transcription factor staining kit was used (eBioscience). For flow cytometry of cultured cancer cells, cells were detached using 0.05% Trypsin and inhibited in full media. For bone marrow derived dendritic cells supernatants was collected.
Instrument	LSR Fortessa (BD)<br>FACS Aria (BD)
Software	FACS Diva (version 9.0.1), FlowJo (version 10.8.0)
Cell population abundance	For sorting CD45+ immune cells, cells were reanalyzed after sorting and a purity of >90% was confirmed.
Gating strategy	For all gating strategies, singlets were identified based on FSC-A/FSC-H. Live dead exclusion was performed by staining with the fixable viability dye eF780 (1:1000, eBioscience). DCs were defined in most experiments as MHCII+ CD11c+ CD24+ out of alive CD45+ cells. cDC1s were identified as CD103+ CD11b- out of the total DCs, cDC2s as CD103- CD11b+ and inflammatory cDC2 as CD103- CD11b+ AXL+. AXL was previously described to identify inflammatory cDC2s. Monocytes were defined as Ly6C+ CD11b+ F4/80-, and inflammatory monocytes were identified as monocytes that were Ly6A+. Ly6A was previously described to identify monocytes expressing high levels of interferon-stimulated genes (ISGs). Macrophages were defined as Ly6C- F4/80+ Cd11b+.For the identification of adoptively transferred CD8+ T cells, CD3+ and CD8+ was used. On this populations we assessed PD-1, TIM-3 and TCF1 levels

☒ Tick this box to confirm that a figure exemplifying the gating strategy is provided in the Supplementary Information.
