# Supplementary material for: Cancer cells impair monocyte-mediated T cell stimulation to evade immunity
Source: Nature. 2024 Nov 27;637(8046):716–25. doi: 10.1038/s41586-024-08257-4 (PMC7617236; doi:10.1038/s41586-024-08257-4)
Supplement: Supplementary file 4 — Supplementary Tables 1–8. [file 41586_2024_8257_MOESM4_ESM.zip › 2023-08-14780D-s4/supplementary-table-legends.docx]

**Supplementary Table Legends:**

**Supplementary Table 1**: **Top 20 markers from scRNA-seq populations**. Gene lists depicting the top differentially expressed genes of each identified population in the scRNA-seq from the immune TME of YUMM1.7^OVA^ tumors.

**Supplementary Table 2**: **scRNA-seq gene signatures**. Publicly available T cell signatures used to probe the scRNA-seq data of OT-1 T cells isolated from YUMM1.7^OVA^ tumors, publicly available inflammatory gene signatures and the Monocyte 1 and Inflammatory Monocyte gene signatures generated with the scRNA-seq dataset from our study.

**Supplementary Table 3**: **Differentially expressed genes in N^TT^ and R^TT^ cancer cells.** Differentially expressed genes (log2FC > 2, p-value < 0.05) from YUMM1.7^OVA^ N^TT^ and R^TT^ cancer cells sorted from tumors and analyzed by RNA-sequencing.

**Supplementary Table 4**: **Differentially expressed genes in the TME and TME signatures.** Gene lists depicting the differentially expressed genes in the immune TME of *Ptgs1/2* KO and IRF3/7 R^TT^ compared to CTRL R^TT^ YUMM1.7^OVA^ tumors (log2FC > 1, p-value < 0.05) analyzed by scRNA-seq. Gene signatures derived (TME-COX and TME-IRF3/7) and their human orthologs are included.

**Supplementary Table 5: Meta-analysis studies.** Main characteristics of studies included in the meta-analysis, including cancer type, numbers of patients, COX inhibitors used and immunotherapy specification.

**Supplementary Table 6: Search query for meta-analysis.** Contains the exact search query that was used for the meta-analysis in this study.

**Supplementary Table 7: RT-qCPR primer and sgRNA list.** Contains a list with all primer pairs used for RT-qPCR experiments and sgRNAs used for CRISPR/Cas9 knockouts.

**Supplementary Table 8: Antibody list.** Contains a list with all antibodies used in this study for flow cytometry, immunofluorescence, western-blotting and *in vivo* and *in vitro* depletion experiments.
